# Detection of group A streptococcus in children with confirmed viral pharyngitis and antiviral host response

**DOI:** 10.1007/s00431-022-04633-2

**Published:** 2022-09-27

**Authors:** Lauri Ivaska, Jussi Niemelä, Kirsi Gröndahl-Yli-Hannuksela, Niina Putkuri, Jaana Vuopio, Tytti Vuorinen, Matti Waris, Kaisu Rantakokko-Jalava, Ville Peltola

**Affiliations:** 1 Departments of Paediatrics and Adolescent Medicine, Turku University Hospital and University of Turku, Turku, Finland; 2 Emergency Services, Turku University Hospital and University of Turku, Turku, Finland; 3 Medical Microbiology and Immunology, Institute of Biomedicine, University of Turku, Turku, Finland; 4 Division of Clinical Microbiology, Turku University Hospital, Turku, Finland; 5 Finnish Red Cross, Blood Service, Helsinki, Finland

**Keywords:** GAS, Group A streptococcus, MxA, Myxovirus resistance protein A, Pharyngitis, *Streptococcus pyogenes*

## Abstract

Our aim was to study the detection of group A streptococcus (GAS) with different diagnostic methods in paediatric pharyngitis patients with and without a confirmed viral infection. In this prospective observational study, throat swabs and blood samples were collected from children (age 1–16 years) presenting to the emergency department with febrile pharyngitis. A confirmed viral infection was defined as a positive virus diagnostic test (nucleic acid amplification test [NAAT] and/or serology) together with an antiviral immune response of the host demonstrated by elevated (≥ 175 µg/L) myxovirus resistance protein A (MxA) blood concentration. Testing for GAS was performed by a throat culture, by 2 rapid antigen detection tests (StrepTop and mariPOC) and by 2 NAATs (Simplexa and Illumigene). Altogether, 83 children were recruited of whom 48 had samples available for GAS testing. Confirmed viral infection was diagnosed in 30/48 (63%) children with febrile pharyngitis. Enteroviruses 11/30 (37%), adenoviruses 9/30 (30%) and rhinoviruses 9/30 (30%) were the most common viruses detected. GAS was detected by throat culture in 5/30 (17%) with and in 6/18 (33%) patients without a confirmed viral infection. Respectively, GAS was detected in 4/30 (13%) and 6/18 (33%) by StrepTop, 13/30 (43%) and 10/18 (56%) by mariPOC, 6/30 (20%) and 9/18 (50%) by Simplexa, and 5/30 (17%) and 6/18 (30%) patients by Illumigene.

*Conclusion*: GAS was frequently detected also in paediatric pharyngitis patients with a confirmed viral infection. The presence of antiviral host response and increased GAS detection by sensitive methods suggest incidental throat carriage of GAS in viral pharyngitis.

**Table:** 

**What is Known:** *•The frequency and significance of GAS-virus co-detection are poorly characterised in children with pharyngitis.* *•Detection of a virus and the antiviral host response likely indicates symptomatic infection.*	
**What is New:** *•Group A streptococcus (GAS) was detected in 17–43% of the children with confirmed viral pharyngitis depending on the GAS diagnostic method.* *•Our results emphasize the risk of detecting and treating incidental pharyngeal carriage of GAS in children with viral pharyngitis.*

**What is Known:**

*•The frequency and significance of GAS-virus co-detection are poorly characterised in children with pharyngitis.*

*•Detection of a virus and the antiviral host response likely indicates symptomatic infection.*

**What is New:**

*•Group A streptococcus (GAS) was detected in 17–43% of the children with confirmed viral pharyngitis depending on the GAS diagnostic method.*

*•Our results emphasize the risk of detecting and treating incidental pharyngeal carriage of GAS in children with viral pharyngitis.*

## Introduction

Acute pharyngitis caused by group A streptococcus (GAS, *Streptococcus pyogenes*) is a common reason for antibiotic prescribing [1, 2]. It has been estimated that the proportion of pharyngitis cases caused by GAS is 24–37% in children [3, 4] and that pharyngitis is associated with substantial antibiotic overuse [5]. Furthermore, only few studies have incorporated modern virological methods [6, 7], and the possibility of a GAS-virus co-detection in children with pharyngitis has thus been overlooked.

The clinical significance of the GAS-virus co-detection in pharyngitis patients is unclear. This is a result of the relatively high asymptomatic oropharyngeal carriage rate of GAS and the frequent detection of respiratory viruses by nucleic acid amplification tests (NAATs) in asymptomatic subjects [3, 4, 8]. We have recently shown that finding a virus in children with febrile pharyngitis is often accompanied by an antiviral host response demonstrated by an elevated blood myxovirus resistance protein A (MxA) level, suggesting a causative role for a virus in these children [7]. An MxA response is mediated by type I and III interferons and correlates with fever and other symptoms [9]. In addition, the emerging use of novel, highly sensitive rapid antigen detection tests (RADTs) and NAATs could further increase the detection rate of GAS [10, 11]. Therefore, we hypothesized that GAS can be detected in part of the children with confirmed viral pharyngitis, likely reflecting an asymptomatic pharyngeal carriage.

### Objective

We used culture, two RADTs and two NAATs to study the occurrence of GAS in paediatric pharyngitis patients with or without a viral infection confirmed by both a positive virus detection and an antiviral MxA response. We postulate that the symptoms in children with a viral infection fulfilling this definition are mostly caused by a virus and not by GAS.

## Materials and methods

This was part of a prospective observational study on the aetiology and diagnosis of febrile pharyngitis in children and adolescents [7]. The study was done at the Department of Emergency Services of Turku University Hospital, Turku, Finland, between November 25, 2013, and January 31, 2015. Inclusion criteria were age 1–16 years, fever during the acute illness episode and acute pharyngitis defined as oropharyngeal exudate and/or intensive redness. Pharyngitis was diagnosed, and a treatment decision was made by the attending emergency department (ED) physician. In the original cohort, the study size was estimated to be sufficient to demonstrate the wide spectrum of viral agents capable of causing pharyngitis. All patients were invited to a follow-up visit 2–4 weeks after the initial visit to collect paired serum samples. In this study, only children with diagnostic test results available by both GAS RADT methods and by both GAS NAAT methods were included.

Confirmed viral infection was defined as a positive virus diagnostic test (NAAT and/or serology) together with the antiviral immune response of the host demonstrated by an elevated (≥ 175 µg/L) blood MxA concentration. The threshold was chosen based on the findings in an earlier study where a blood MxA concentration ≥ 175 µg/L best differentiated symptomatic viral infections from asymptomatic conditions in children [9].

The Ethics Committee of the Hospital District of Southwest Finland approved the study protocol. The legal guardians of all participating children and adolescents and the adolescent patients themselves gave their written, informed consent.

### Sample collection and GAS RADTs

At the enrolment, blood samples for biomarker measurement and serology and oropharyngeal swab samples for culture, RADT and NAATs were collected. From each child, four throat swabs were collected in a standardized order by rubbing the swabs against both tonsils: (1) A flocked swab was used to collect the sample for bacterial culture and transferred in a tube with liquid transport media (ESwab, Copan, Brescia, Italy) to the Division of Clinical Microbiology, Turku University Hospital for bacterial culture and GAS NAAT analysis. (2) A flocked swab (Copan) was used to collect the sample for virus NAAT and transferred in a dry, clean tube to the Laboratory of Diagnostic Virology of Turku University Hospital for analysis. (3) The swab sample for an immunochromatographic GAS RADT was collected, and the test was performed immediately at the ED according to the manufacturer’s instructions (StrepTop, AllDiag, Strasbourg, France). (4) The sample for automated GAS RADT was collected by a flocked swab (Copan) and transferred in a dry, clean tube to the Central Laboratory of Turku University Hospital, where the test was immediately performed according to the manufacturer’s instructions (mariPOC, ArcDia International Ltd., Turku, Finland).

### Throat culture, virus diagnostics and biomarker measurements

Bacterial culture, virus NAAT and serology and blood MxA measurements were performed as described in more detail earlier [7]. Briefly, throat culture for GAS was performed by inoculating transport media on streptococcal selective blood agar plate including colistin-oxolinic acid, as well as in a streptococcal-selective broth, followed by subculture on streptococcal-selective blood agar. Beta-haemolytic streptococci were identified by standard methods, including the Lancefield antigen test (PathoDxtra Strep Grouping Kit, Thermo Scientific, Basingstoke, UK) and/or matrix-assisted laser desorption and ionization time-of-flight mass spectrometry (Bruker Daltonics, Bremen, Germany). Respiratory virus diagnostics were performed by a multiplex respiratory virus real-time–reverse-transcription polymerase chain reaction (RT–PCR) test (Anyplex II RV16; Seegene, Seoul, Korea), by an in-house–real-time RT–PCR assay for rhinoviruses and enteroviruses and by serological methods (Epstein-Barr virus, adenovirus, enteroviruses, influenza A and B viruses, parainfluenza virus types 1, 2 and 3 and respiratory syncytial virus) mainly from paired serum samples [7]. Plasma C-reactive protein (CRP) and procalcitonin levels were determined in the hospital central laboratory by routine methods. Whole blood samples for MxA measurement were transported to the laboratory where samples were diluted 1:20 in hypotonic buffer and stored at −70 °C until the enzyme immunoassay analysis was performed as described earlier [9, 12, 13].

### GAS serology

Anti-streptolysin O (ASO) antibody levels in serum were determined using the RapiTex ASL kit (Siemens Healthcare Diagnostics Products GmbH, Marburg, Germany) according to the manufacturer’s instructions.

### GAS NAATs

Molecular methods tested were Simplexa GAS assay using 3 M Integrated Cycler (Focus Diagnostics, Cypress, CA, USA) and Illumigene GAS assay (Meridian Bioscience, Paris, France). Tests were performed as per the manufacturer’s instructions except that the swab samples were replaced with 50 µl of the Eswab transport media.

### Data analysis

A confirmed viral infection was defined as a positive virus detection (NAAT and/or serology) in a child with a documented antiviral innate immune response (blood MxA concentration ≥ 175 µg/L). Sensitivities, specificities, positive and negative predictive values and test accuracies with 95% confidence intervals (CI) were calculated for each GAS diagnostic assay in comparison with the throat culture. The percentage of the GAS detection rate and the occurrence of virus co-detection and elevated blood MxA levels in GAS-positive pharyngitis patients with 95% CIs were calculated separately for each GAS diagnostic assay. Nominal data were analysed by χ^2^ or Fisher’s exact tests. IBM SPSS Statistics, version 27 (IBM Corp., Armonk, NY, USA) was used for analysis.

## Results

In total, 83 children (median age 5.5 years, interquartile range (IQR) 3.2–12.2) with febrile pharyngitis were recruited. The final study population included 48 children (median age 8.2 years, IQR 3.4–12.5) with specimens available for GAS RADT and NAAT analysis by all four methods. Throat culture was positive for GAS in 11/48 (23%) of the final study population and in 19/83 (23%) of all recruited children, showing that our study sample was comparable to the larger patient population with febrile pharyngitis in terms of GAS occurrence.

Viruses were detected in 36/48 (75%), elevated blood MxA levels in 36/48 (75%) and both in 30/48 (63%) children, comprising the group of confirmed viral infection. Enteroviruses, adenoviruses and rhinoviruses were the most frequently detected viruses. More than 1 virus was detected in 13/48 (27%) children. All virus findings are presented in detail in Table 1. Cough and/or rhinitis were present in 27/48 (56%) children. Median (IQR) C-reactive protein (CRP) plasma levels in children with confirmed and no confirmed viral infection were 10 (5–14) mg/L and 34 (10–45) mg/L, and procalcitonin levels were 0.1 (0.1–0.3) µg/L and 0.1 (0.1–0.3) µg/L, respectively. When CRP results were combined with MxA results as a MxA (µg/L)/CRP (mg/L) ratio, the median (IQR) levels were in the group of confirmed viral aetiology 97 (65–187) and in the group of no confirmed viral aetiology 6 (3–21).

**Table 1:** Virus detection in pharyngitis patients with and without demonstrable antiviral host response

	**Blood MxA concentration ≥ 175 µg/L (n = 36)**		**Blood MxA concentration < 175 µg/L (*n* = 12)**	
Virus detection positive, *n* (%)	30 (83)		6 (50)	
Virus findings, *n*	Rhinovirus Influenza A virus Adenovirus Enterovirus Human metapneumovirus Parainfluenza virus 3 Adenovirus and human coronavirus OC43 Epstein-Barr virus Respiratory syncytial virus B Influenza A virus and adenovirus Enterovirus and parainfluenza virus 3 Enterovirus, parainfluenza virus 3, and rhinovirus Enterovirus and rhinovirus Enterovirus and parainfluenza virus 2 Human metapneumovirus and enterovirus Enterovirus and adenovirus Adenovirus, enterovirus, and rhinovirus Enterovirus, adenovirus, and rhinovirus Adenovirus and rhinovirus Respiratory syncytial virus A, adenovirus, enterovirus, and human coronavirus OC43	4 3 2 2 2 2 1 1 1 1 1 1 1 1 1 1 1 1 1 1	Human coronavirus NL63 Rhinovirus Bocavirus and rhinovirus Enterovirus	2 2 1 1
No viruses detected, *n* (%)	6 (17)		6 (50)	

*MxA* myxovirus resistance protein A

GAS was detected by throat culture in 5/30 (17%) with and in 6/18 (33%) without confirmed viral infection (*p* = 0.288). The detection rate of GAS (proportion of positive samples of all tested) was lower among children with confirmed viral infection than among children without it, regardless of the GAS test (Table 2). ASO antibodies were detected at enrolment in 1/5 (20%) children with confirmed viral infection and GAS-positive throat culture and in 2/6 (33%) without confirmed viral infection and GAS positive throat culture. Paired serum samples were collected from 31/48 (65%) children for a median of 18.5 days (IQR 17–21) apart. Only one child showed ≥ fourfold increase in ASO levels and in this case throat culture was negative for GAS.

**Table 2:** Group A streptococcus (GAS) test results of 48 children with pharyngitis with and without confirmed viral infection

	**Confirmed viral infection (*n* = 30)**^**a**^**, GAS detected**		**No confirmed viral infection (*n* = 18)**^**a**^**, GAS detected**		
**GAS diagnostic method**	***n***	**% (95% CI)**	***n***	**% (95% CI)**	***P***^**b**^
Throat culture	5	17 (6–27)	6	33 (20–47)	0.288
StrepTop	4	13 (4–23)	6	28 (15–40)	0.265
mariPOC	13	43 (29–57)	10	56 (41–70)	0.412
Simplexa	6	20 (9–31)	9	50 (36–64)	0.030
Illumigene	5	17 (6–27)	6	33 (20–47)	0.288

*CI* confidence interval

^a^Confirmed viral infection: virus detected and blood myxovirus resistance protein A concentration ≥ 175 µg/L

^b^χ^2^ or Fisher’s exact test

When the diagnostic performances of GAS RADTs and NAATs were compared with throat culture, distinct test features could be seen; StrepTop (an immunochromatographic RADT) had a sensitivity of 82% and a specificity of 100%. MariPOC (an automated RADT) had a 100% sensitivity and 68% specificity. The two GAS NAATs Simplexa and Illumigene had a sensitivity of 91% both and a specificity of 86% and 97%, respectively. In this study, the overall accuracy was highest with StrepTop and Illumigene assays (Table 3).

**Table 3:** Diagnostic performance of four different group A streptococcus (GAS) assays in comparison with throat culture (n = 48)

		**Performance, % (95% CI)**				
	**GAS positive samples, *n* (%)**	**Sensitivity**	**Specificity**	**PPV**	**NPV**	**Accuracy**^**a**^
**Throat culture**	11 (23)					
**RADTs**						
StrepTop	9 (19)	82 (71–93)	100	100	95 (89–100)	96 (90–100)
mariPOC	23 (48)	100	68 (54–81)	48 (34–62)	100	75 (63–87)
**NAATs**						
Simplexa	15 (31)	91 (83–99)	86 (77–96)	67 (53–80)	97 (92–100)	88 (78–97)
Illumigene	11 (23)	91 (83–99)	97 (93–100)	91 (8–99)	97 (93–100)	96 (90–100)

*CI* confidence interval *PPV* positive predictive value, *NPV* negative predictive value, *RADT* rapid antigen detection test, *NAAT* nucleic acid amplification test

^a^Accuracy = the percentage of correctly classified samples

## Discussion

The most important finding in this study is that GAS can be frequently detected also in children with pharyngitis of a confirmed viral aetiology. Depending on the diagnostic method, GAS was identified in 13–43% of them. By using the novel concept of confirmed viral infection defined as a virus finding and an antiviral immune response of the host as indicated by elevated blood MxA level, we aimed to minimize the risk of labelling clinically insignificant virus findings by NAAT as true infections. Blood MxA level increases due to type I and III interferon production as part of the innate immune response triggered by a symptomatic viral infection. Therefore, MxA is a promising and a relatively simple biomarker that has proven to be a useful addition to virological methods in epidemiological or diagnostic studies [7, 9, 14–16].

Some pre-clinical and ex-vivo data suggest that also GAS infection could trigger type I interferon response [17–19]. However, the role of interferon production in clinical GAS pharyngitis remains unknown. In our cohort, GAS was detected by throat culture in 11 children. Any virus was co-detected in 6/11 (55%) of these GAS-infected patients. Blood MxA level was elevated (≥ 175 µg/L) in 5/6 (83%) of these patients and in none of the GAS positive but virus negative (*n* = 5) patients. This preliminary finding suggests that GAS pharyngitis without concomitant viral infection does not induce a systemic interferon response that would complicate the use of blood MxA level as a biomarker for acute viral infection. Furthermore, earlier data on CRP in differentiating GAS from other aetiologies of pharyngitis does not support its use [21, 22]. Our data on combining viral and bacterial biomarkers in pharyngitis patients is promising and in line with our recent findings in hospitalized febrile children [20].

There were 6/36 (17%) children with high blood MxA levels (≥ 175 µg/L) without any viruses detected. The reason for this can only be speculated, but it seems possible that some of them had a viral infection that we were not able to diagnose. There were 6/12 (50%) children with virus finding among those with low blood MxA levels (< 175 µg/L). As stated before, viruses can often be detected also in asymptomatic children by NAAT methods [8]. In these asymptomatic infections, host response, including interferon/MxA response, differs from that of symptomatic infection [9, 20]. This could be the case also with the virus-positive but MxA-negative children in our cohort.

Our results are in line with another pragmatic study in children with pharyngitis by Shapiro et al. [21]. They found that 53% of the children diagnosed with GAS pharyngitis had at least one clinical sign or symptom (cough, rhinitis, ulcers/vesicles on their mucosa or conjunctival injection) suggestive of viral infection. In our study, 5/11 (45%) of the children with throat culture-positive GAS pharyngitis had a confirmed viral infection. Thus, our study with a comprehensive virological workup completes the earlier clinical findings and confirms the role of viruses as the most probable cause of illness in children with GAS-virus co-detection. In children from high-income countries, the rate of asymptomatic pharyngeal GAS carriage is estimated to be around 10% [3, 4]. In other words, detection of GAS most likely reflects an asymptomatic pharyngeal carriage and contributes to the risk of unnecessary antibiotic prescribing.

Another notable observation is the variation in the sensitivity and specificity of different diagnostic GAS assays in comparison with the throat culture. All four assays showed relatively good sensitivity, 82–100%, but the specificity between different assays was more variable, 68–100%. The exceptionally high detection rate of mariPOC GAS RADT is in line with an earlier study of this assay [10]. Technology in GAS NAATs is based either on RT-PCR, such as Simplexa GAS assay or isothermal amplification, such as Illumigene GAS assay. In settings where the burden of rheumatic fever and other post-streptococcal sequel is high, antibiotic treatment is recommended for all suspected GAS infections. In these settings, highly sensitive GAS assays could reduce unnecessary antibiotic use [22]. In other settings, the potential impact of the increased analytical sensitivity of GAS NAATs on the antimicrobial prescription rate for pharyngitis is of concern [11, 23]. All these findings emphasize the importance of proper validation of novel diagnostic methods and highlight the role of clinical guidelines in use of them.

Our study is subject to limitations. Due to the small size of the study population, our findings should be interpreted as preliminary observations. It is also worth noticing that stringent diagnostic criteria for pharyngitis were not used in this study. Thus, children with symptoms suggestive of viral infection were not categorically excluded if the attending ED physician diagnosed acute pharyngitis. Our study design was pragmatic and based on how pharyngitis is diagnosed, and how these children are treated at the actual site of care. Furthermore, the collection of four throat swabs might theoretically influence the results despite the standardised sampling protocol.

## Conclusions

Our data show that GAS can be frequently detected in paediatric pharyngitis patients with a viral infection confirmed by microbiological diagnosis together with the demonstration of an antiviral innate immune response. When the GAS diagnosis was based on a highly sensitive assay instead of the throat culture, co-detections were even more frequent. These findings emphasize the risk of detecting and treating asymptomatic pharyngeal carriage of GAS, highlight the importance of diagnostic stewardship in children with pharyngitis and call for larger studies to examine the GAS-virus co-detections.

## Data Availability

The datasets generated during the current study are not publicly available due to privacy protection but are available from the corresponding author on a reasonable request.

## Abbreviations

ASO: Anti-streptolysin O
CI: Confidence interval
CRP: C-reactive protein
ED: Emergency department
GAS: Group A streptococcus
IQR: Interquartile range
MxA: Myxovirus resistance protein A
NAAT: Nucleic acid amplification test
RADT: Rapid antigen detection test
RT-PCR: Reverse-transcription polymerase chain reaction

## References

[1] Fleming-Dutra KE, Hersh AL, Shapiro DJ (2016). Prevalence of inappropriate antibiotic prescriptions among US ambulatory care visits, 2010–2011. JAMA.

[2] Lewnard JA, King LM, Fleming-Dutra KE (2020). Incidence of pharyngitis, sinusitis, acute otitis media, and outpatient antibiotic prescribing preventable by vaccination against group A Streptococcus in the United States. Clin Infect Dis.

[3] Shaikh N, Leonard E, Martin JM (2010). Prevalence of streptococcal pharyngitis and streptococcal carriage in children: a meta-analysis. Pediatrics.

[4] Oliver J, Malliya Wadu E, Pierse N (2018). Group A Streptococcus pharyngitis and pharyngeal carriage: a meta-analysis. PLoS Negl Trop Dis.

[5] Dooling KL, Shapiro DJ, Van Beneden C (2014). Overprescribing and inappropriate antibiotic selection for children with pharyngitis in the United States, 1997–2010. JAMA Pediatr.

[6] Mistik S, Gokahmetoglu S, Balci E, Onuk FA (2015). Sore throat in primary care project: a clinical score to diagnose viral sore throat. Fam Pract.

[7] Ivaska L, Niemelä J, Lempainen J (2017). Aetiology of febrile pharyngitis in children: potential of myxovirus resistance protein A (MxA) as a biomarker of viral infection. J Infect.

[8] Jartti T, Jartti L, Peltola V (2008). Identification of respiratory viruses in asymptomatic subjects: asymptomatic respiratory viral infections. Pediatr Infect Dis J.

[9] Toivonen L, Schuez-Havupalo L, Rulli M (2015). Blood MxA protein as a marker for respiratory virus infections in young children. J Clin Virol.

[10] Vakkila J, Koskinen JO, Brandt A (2015). Detection of group A Streptococcus from pharyngeal swab samples by bacterial culture is challenged by a novel mariPOC point-of-care test. J Clin Microbiol.

[11] Tanz RR, Zheng XT, Carter DM (2018). Caution needed: molecular diagnosis of pediatric group A Streptococcal pharyngitis. J Pediatric Infect Dis Soc.

[12] Vallittu a-M, Erälinna J-P, Ilonen J,  (2008). MxA protein assay for optimal monitoring of IFN-beta bioactivity in the treatment of MS patients. Acta Neurol Scand.

[13] Maria NI, Brkic Z, Waris M (2014). MxA as a clinically applicable biomarker for identifying systemic interferon type I in primary Sjogren’s syndrome. Ann Rheum Dis.

[14] Engelmann I, Dubos F, Lobert P-E (2015). Diagnosis of viral infections using myxovirus resistance protein A (MxA). Pediatrics.

[15] Piri R, Ivaska L, Yahya M (2020). (2020) Prevalence of respiratory viruses and antiviral MxA responses in children with febrile urinary tract infection. Eur J Clin Microbiol Infect Dis.

[16] Piri R, Yahya M, Ivaska L (2022). Myxovirus resistance protein A as a Marker of viral cause of illness in children hospitalized with an acute infection. Microbiol Spectr.

[17] Gratz N, Hartweger H, Matt U (2011). Type I interferon production induced by Streptococcus pyogenes-derived nucleic acids is required for host protection. PLoS Pathog.

[18] Hyland KA, Brennan R, Olmsted SB (2009). The early interferon response of nasal-associated lymphoid tissue to Streptococcus pyogenes infection. FEMS Immunol Med Microbiol.

[19] Miettinen M, Veckman V, Latvala S (2008). Live Lactobacillus rhamnosus and Streptococcus pyogenes differentially regulate Toll-like receptor (TLR) gene expression in human primary macrophages. J Leukoc Biol.

[20] Heinonen S, Jartti T, Garcia C (2016). Rhinovirus detection in symptomatic and asymptomatic children: value of host transcriptome analysis. Am J Respir Crit Care Med.

[21] Shapiro DJ, Lindgren CE, Neuman MI, Fine AM (2017). Viral features and testing for streptococcal pharyngitis. Pediatrics.

[22] Ralph AP, Holt DC, Islam S (2019). Potential for molecular testing for group a streptococcus to improve diagnosis and management in a high-risk population: a prospective study. Open Forum Infect Dis.

[23] Jaggi P, Leber A (2021). Molecular testing for group a streptococcal pharyngitis: to test or not to test, that is the question. J Pediatric Infect Dis Soc.

